# Is the left insular cortex associated with the exaggerated activity in the parasympathetic nervous system?

**DOI:** 10.1016/j.cnp.2021.03.003

**Published:** 2021-03-31

**Authors:** Michiaki Nagai, Masaya Kato, Keigo Dote

**Affiliations:** Department of Cardiology, Hiroshima City Asa Hospital, Hiroshima 731-0293, Japan

We have read the case report by Wali and Siddiqui (2021) about a patient with epilepsy and focal cortical dysplasia including the insular cortex (Ic) in relation to prolonged ictal asystole. They suggested that the abnormal Ic activity was associated with autonomic instability underlying the pathophysiology of adverse effects on the patient's cardiovascular system. We note that several points should be taken into consideration to understand the etiology of the patient described by Wali and Siddiqui (2021).

Our earlier research supported the notion that the human cardiovascular system is regulated by a cortical network consisting of the anterior cingulate gyrus, amygdala, and Ic (Nagai et al., 2010, Nagai et al., 2017). In patients with drug-refractory epilepsy, right hemispheric inactivation was shown to increase the high-frequency power of the heart rate and blood pressure, and left hemispheric inactivation was shown to increase the low-frequency power of the heart rate and blood pressure (Hilz et al., 2001). A cardiac representation site was also identified in the human Ic (Oppenheimer et al., 1992). In that study, tachycardia or pressor effects were more commonly observed after stimulation of the right Ic, and bradycardia or depressor effects were more commonly observed after stimulation of the left Ic. The right Ic is involved in the activity of the sympathetic nervous system, and the left Ic is involved in the activity of the vagal nervous system. A functional MRI study indicated that the sympatho-vagal balance is dependent on the integrity of the lateralized salience network including the left Ic for the vagal nervous system (Sturm et al., 2018). In the patient described by Wali and Siddiqui et al. (2021), electrocardiography demonstrated that the baseline tachycardia changed to bradycardia, followed by cardiac asystole. Thus, ictal activity might spread from the right hemisphere to the left Ic prior to the occurrence of asystole.

The relationship between Ic activity and the sympatho-vagal balance according to the hemispheric laterality has been rarely assessed in patients with epilepsy. Further studies may help elucidate the mechanisms underlying this relationship.

## Disclosure

The authors state that they have no conflicts of interest.
